# The Impact Of A Whole Health Model Of Care On Patient Outcomes

**DOI:** 10.1093/geroni/igab046.3048

**Published:** 2021-12-17

**Authors:** Anna Faul, Pamela Yankeelov, Joseph D'Ambrosio, Sam Cotton, Barbara Gordon, Maria Hall-Faul

**Affiliations:** 1 University of Louisville, Louisville, Kentucky, United States; 2 University of Louisville, University of Louisville, Kentucky, United States; 3 University of Louisville Trager Institute, University of Louisville Trager Institute, Louisville KY, Kentucky, United States; 4 University of Connecticut, University of Connecticut, Connecticut, United States

## Abstract

The FlourishCare Model (FCM) transforms primary care sites by addressing all determinants of health and focusing on helping patients to flourish. The FlourishCare Index (FCI) is a clinical measure to assess the effectiveness of the FCM to address all determinants of health. We will present data on the effectiveness of the FCM serving 159 older adults with MCCs. The sample was mostly female (77%), White (64%), retired (54%), married (30%) or widowed (20%) and living in urban areas (64%). The mean age was 69 (SD=15), with 13 years education (SD=3). Patients changed significantly over time on total FCI scores (57%-72%;□=3.80,SE=0.63). Results show significant growth over time for individual health behaviors (58%-67%;□=2.14,SE=0.84), health care access (71%-89%;□=4.43,SE=1.00) and social determinants (62%-85%;□=5.54,SE=1.02) with psychological determinants (54%-61%;□=1.74,SE=0.95) and environmental determinants (70%-81%;□=2.81,SE=1.62) showing a trend to significance. Interaction effects with time show that the FCM supported patients with lower education attainment to improve at a higher rate than those with higher education attainment, for the total FCI score (□=-0.59,SE=0.24) and health care access (□=-0.94,SE=0.38). Receiving mental health counseling resulted in more improvement in psychological determinants than those who did not receive counseling (□=3.43,SE=2.04). The FCM was able to support rural patients at a higher rate than urban patients to gain access to health care (□=4.13,SE=2.02). The FCM supported Hispanic patients the most in improving social determinants of health (□=8.40,SE=3.93). This study showed the importance of a systems approach to care using measures that focus on what matters most to older adults who value quality-of-life outcomes.

